# Errata

**Published:** 1895-02

**Authors:** 


ERRATA.—In December issue, page 601, one line was left out by the printer. Five lines from the bottom should read, "The patient presented in March, 1821. After an examination, Dr. Antony made a diagnosis of empyema, and concluded," etc. In the January issue Dr. Hagan's excellent article on Migraine was not properly read in proof, through a misunderstanding with the author. On page 663, second line, *cadage* should be *lavage;* in sixth line *grains* ten to fifteen should be *drops*, etc.; in seventh line *one grain* should be *one dram;* in tenth line, for *beneficial in sea travel* read *beneficial; also sea travel;* in eighth line *Bland* should be *Blaud.* On page 681, twelfth line, *bi-monthly meetings* should be *bi-weekly.*

